# Oxygen transfer rate identifies priming compounds in parsley cells

**DOI:** 10.1186/s12870-015-0666-3

**Published:** 2015-11-25

**Authors:** Jana Viola Schilling, Britta Schillheim, Stefan Mahr, Yannik Reufer, Sandi Sanjoyo, Uwe Conrath, Jochen Büchs

**Affiliations:** AVT – Biochemical Engineering, RWTH Aachen University, Worringer Weg 1, D-52074 Aachen, Germany; Department of Plant Physiology, RWTH Aachen University, Worringer Weg 1, D-52074 Aachen, Germany

**Keywords:** Parsley cell culture, Priming, Salicylic acid, Pep13, Oxygen transfer rate, Respiration activity monitoring system (RAMOS)

## Abstract

**Background:**

In modern agriculture, the call for an alternative crop protection strategy increases because of the desired reduction of fungicide and pesticide use and the continuously evolving resistance of pathogens and pests to agrochemicals. The direct activation of the plant immune system does not provide a promising plant protection measure because of high fitness costs. However, upon treatment with certain natural or synthetic compounds, plant cells can promote to a fitness cost-saving, primed state of enhanced defense. In the primed state, plants respond to biotic and abiotic stress with faster and stronger activation of defense, and this is often associated with immunity and abiotic stress tolerance. Until now, the identification of chemical compounds with priming-inducing activity (so-called plant activators) relied on tedious and invasive approaches, or required the late detection of secreted furanocoumarin phytoalexins in parsley cell cultures. Thus, simple, fast, straightforward, and noninvasive techniques for identifying priming-inducing compounds for plant protection are very welcome.

**Results:**

This report demonstrates that a respiration activity-monitoring system (RAMOS) can identify compounds with defense priming-inducing activity in parsley cell suspension in culture. RAMOS relies on the quasi-continuous, noninvasive online determination of the oxygen transfer rate (OTR). Treatment of parsley culture cells with the known plant activator salicylic acid (SA), a natural plant defense signal, resulted in an OTR increase. Addition of the defense elicitor Pep13, a cell wall peptide of *Phythophthora sojae*, induced two distinctive OTR peaks that were higher in SA-primed cells than in unprimed cells upon Pep13 challenge. Both, the OTR increase after priming with SA and the Pep13 challenge were dose-dependent. Furthermore, there was a close correlation of a compound’s activity to enhance the oxygen consumption in parsley cells and its capacity to prime Pep13-induced furanocoumarin secretion as evaluated by fluorescence spectroscopy.

**Conclusions:**

RAMOS noninvasively determines the OTR as a measure of the metabolic activity of plant cells. Chemical enhancement of oxygen consumption by salicylic derivatives in parsley cell suspension cultures correlates with the induction of the primed state of enhanced defense that enhances the quantity of Pep13-induced furanocoumarin phytoalexins. Treatment with the priming-active compounds methyl jasmonate and pyraclostrobin also resulted in an enhanced respiration activity. Thus, RAMOS is a novel technology for identifying priming-inducing compounds for agriculture.

**Electronic supplementary material:**

The online version of this article (doi:10.1186/s12870-015-0666-3) contains supplementary material, which is available to authorized users.

## Background

More than ever, plants are essential to provide food, feed, fibers, and bioenergy to an ever-increasing world population. Sustainable and efficient plant protection strategies are needed to ensure sufficient plant production by minimizing yield losses to biotic and abiotic stresses [1]. Conventionally, synthetic fungicides, pesticides, and herbicides are used to protect plants from biotic stress. However, such chemicals frequently also impair non-target organisms, often are poorly degradable, thus accumulating in the crop, soil, or both. In addition, resistances of pests, pathogens, and weeds to the agrochemicals in use continue to evolve [1]. Thus, the need for an alternative crop protection strategy is becoming increasingly important.

An alternative, sustainable crop protection strategy primes the intrinsic plant immune system for enhanced defense to biotic and abiotic stress [2, 3]. In fact, upon treatment with certain natural or synthetic compounds, plants can switch to a primed state of the alert. In the primed state, plants respond to biotic or abiotic challenges with faster and stronger activation of defense, and this often leads to immunity and/or stress tolerance [3–6].

In fact, the commercial success of some agrochemicals (e.g., strobilurin fungicides, neonicotinoid-based insecticides) at least in part relies on their ability to prime plants for enhanced defense. However, reliable test systems for identifying priming-inducing chemical compounds are rare. A recently reported high-throughput assay for immune-priming compounds assesses the enhancement of bacteria-induced cell death in *Arabidopsis* culture cells by priming agents [7]. In addition to bacterial challenge, the test requires Evans blue staining, washing, dye extraction, and extinction measurement. Therefore, the screen is rather tedious.

In 1998, Siegrist et al. [8] reported that a fluorescence assay facilitates the identification of plant immune-priming compounds. The test relies on parsley culture cells and Pep13, a 13 amino-acid defense elicitor of *Phytophthora sojae* [9]. The assay measures the enhancement of Pep13-induced furanocoumarin (phytoalexin) secretion upon pretreatment with priming activators [10]. The advantage of the test over competitive assays is the high sensitivity of furanocoumarin fluorescence detection and the use of only two subsequent treatments (priming, Pep13 challenge) before final analysis. For example, prolonged pretreatment (priming) of the parsley cells with the plant defense signals jasmonic acid (JA), salicylic acid (SA), or the synthetic SA mimic benzothiadiazole (BTH; trade names Bion®, Actigard®, or Boost®) enhanced the Pep13-activated secretion of antimicrobial furanocoumarin phytoalexins as detected by fluorescence spectroscopy [8, 10, 11]. A disadvantage of the test is its dependence of furanocoumarin secretion that is usually determined not until 24 h after Pep13 challenge [10].

In this study, we report that a respiration activity monitoring system (RAMOS) [12, 13] can identify compounds with plant defense-priming activity. RAMOS enables the noninvasive online monitoring of the oxygen transfer rate (OTR) of parsley cells in suspension culture. As the storage capacity of aqueous solutions for oxygen is very small, the OTR can be equaled to the oxygen uptake rate of parsley plant cells. The impact of SA and Pep13 on the OTR was investigated. Furthermore, the impact of known priming-active (SA, 4-CSA, MeJA, F500) and priming-inactive (3-HBA, 4-HBA) compounds was tested.

## Results and discussion

### Transfer of parsley cell cultures to the RAMOS device

The parsley cell culture was transferred to the RAMOS device to develop a simple, reliable assay to screen for defense priming compounds in plants. Previous studies [14–16] already showed that the respiration activity of plant cells gives crucial information about their metabolism.

**Fig. 1:**
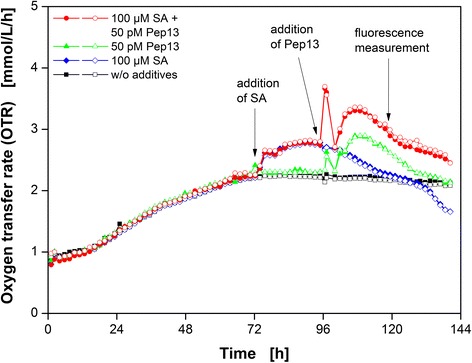
Respiratory response of parsley cell suspension cultures after treatment with the priming compound SA and Pep13. Oxygen transfer rate as a function of time of parsley suspension cultures treated with 100 μM salicylic acid (SA) and 50 pM Pep13 *(red circles)*. Reference cultivations: w/o additives *(black squares)*, with addition of exclusively 100 μM SA *(blue diamonds)*, and with the addition of exclusively 50 pM Pep13 *(green up triangles)*. SA was added after 72 h and Pep13 after 96 h. Cultivation conditions: 250 mL flask volume, 50 mL filling volume, 180 rpm shaking frequency, 50 mm shaking diameter, and 25 °C. Arrows indicate the addition of SA, Pep13, and sampling for the 2D-fluorescence measurements. Experiments were conducted in duplicate *(open and closed symbols)*

First, a standard experiment with priming by SA and subsequent Pep13 challenge [9, 17] was conducted to evaluate the influence of each compound on the OTR of the parsley cells in suspension cultures. An untreated culture without the addition of SA or Pep13, a culture treated exclusively with SA, and a culture treated exclusively with Pep13 were run as references. Figure 1 displays the OTR of parsley cell cultures in these four experiments as a function of time.

All duplicate OTR curves show great consistence (Fig. 1). The OTR curve of the untreated parsley cell culture showed a continuous increase until about 72 h. Addition of 100 μM SA caused a 1.2-fold increase in the OTR compared to the untreated culture at 12 h post SA addition remaining on this level for another 12 h. Addition of exclusively 50 pM Pep13 at 96 h resulted in a first OTR peak with a 1.2-fold increase in the OTR compared to the OTR level of untreated cells (Fig. 1). A second OTR increase started to occur after 100 h. The OTR of the cell culture treated with 100 μM SA and 50 pM Pep13 showed both an increase in OTR upon SA addition and two OTR maxima after the addition of 50 pM Pep13. Both maxima were more pronounced when compared to the culture treated with 50 pM Pep13 exclusively.

Previous studies provided two possible explanations for the enhanced oxygen consumption during priming of plant immunity: One explanation claimed that the activation of mitochondrial alternative oxidase (AOX) might cause enhanced oxygen consumption [18–23], whereas another explanation suggested that the synthesis of reactive oxygen species by the plasma membrane-localized NADPH oxidase caused the enhanced oxygen consumption [18, 24, 25]. The two explanations, though being alternative, are not mutually exclusive, however. Moreover, other oxygen consuming mechanisms in plant defense might be responsible for the observed increase in OTR, for instance the generation of ROS [24, 26–28]. A possible source of O_2_^−^ that is affected by SA is the bona fide electron transport chain located within the mitochondrial membrane [26, 29]. Kawano and Muto (1999) suggested that SA prevents the cell from forming highly reactive hydroxyl radicals (HO^•^). Instead, the tobacco cells produce less reactive O_2_^−^ [30]. The superoxide dismutase (SOD) might further catalyze O_2_^−^ to H_2_O_2_ and SA activates the SOD in tobacco [31]. Additionally, SA inhibits two enzymes which normally degrade H_2_O_2_, catalase and ascorbate peroxidase, resulting in an increase in H_2_O_2_ [24, 27, 28, 32]. Thus, the increase in OTR upon addition of SA could be explained by the activation of several oxygen-consuming mechanisms that in turn might influence the Pep13 response.

Addition of Pep13 initiates the second, pronounced phase of enhanced oxygen consumption in parsley cells, which likely imitates presence of a pathogen. Pep13 and other microbe-associated molecular patterns (MAMPs) activate a myriad of defense responses in plant cells [9]. In previous publications [33, 34], a H_2_O_2_ burst has been described which consists of two phases: a fast, seemingly unspecific generation of ROS followed by a long-lasting, obviously pathogen-specific increase. Nürnberger et al. [9] stated that in parsley cells, Pep13 activates the plasma membrane-bound NAD(P)H oxidase, resulting in O_2_^−^ formation. Subsequently, O_2_^−^ can be converted to H_2_O_2_ and acts as an activator of gene expression in the nucleus [9]. Since oxygen is needed for the formation of O_2_^−^ and H_2_O_2_, the increase in oxygen consumption of parsley cells upon Pep13 addition (Fig. 1) might serve the NAD(P)H-mediated oxidative burst [33, 34].

Pretreatment with SA potentiated the OTR burst induced by Pep13 treatment, as shown in Fig. 1. Both, the first and second OTR peaks were more pronounced compared to the peaks of the culture exclusively treated with Pep13. If the OTR burst reflects the oxidative burst, this potentiated oxygen consumption could be explained by enhanced H_2_O_2_ production [25, 35].

**Fig. 2:**
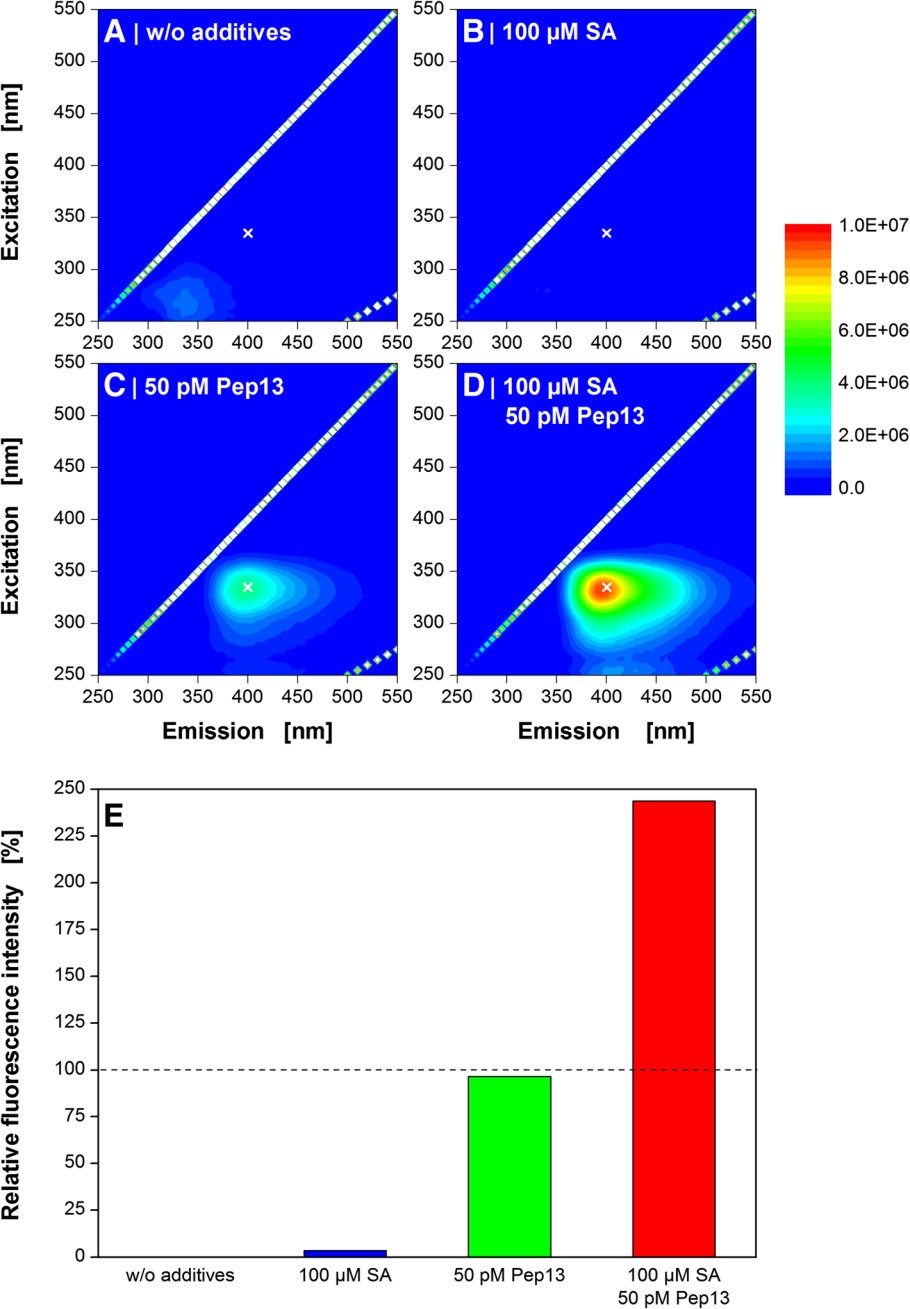
Fluorescence spectroscopy of Pep13-induced furanocoumarins in parsley cell suspension cultures. Cells were treated with the priming compound salicylic acid (SA) after 72 h and with Pep13 after 96 h. Cell culture supernatant was taken 24 h after the addition of Pep13 and subjected to 2D-fluorescence spectroscopy. Experimental conditions: quartz cuvette, 3 mL filling volume, spectral range of 250–550 nm. Reference cultivations: w/o additives (**a**), with the addition of exclusively 100 μM SA (**b**), and with the addition of exclusively 50 pM Pep13 (**c**). The priming experiment includes the addition of 100 μM SA and 50 pM Pep13 (**d**). All experiments are compared in (**e**) at an excitation wavelength of 335 nm and an emission wavelength of 400 nm (marked with a white cross in **a**-**d**). Reference cultivations: w/o additives (*black column*), with the addition of exclusively 100 μM salicylic acid (SA) (*blue column*), and with the addition of exclusively 50 pM Pep13 (*red column*). The total fluorescence signals from the supernatants of the cultures were subtracted by the total fluorescence signal of the untreated culture. Thereupon, the corrected fluorescence intensities of “100 μM SA” (*blue column*) and “50 pM Pep13” *(green column)* were set to 100 %. The priming experiment contained both the addition of 100 μM SA and 50 pM Pep13 (*red column*)

The qualitative determination of secreted furanocoumarin phytoalexins by fluorescence measurement had already been established for detection of defense priming [8, 10, 36]. Fluorescence measurements at a single wavelength pair (λ_exc_ = 335 nm and λ_em_ = 398 nm) were extended to 2D-fluorescence measurements (Fig. 2). A possible shift in the intensity maximum due to minor changes in the furanocoumarin composition can be detected by 2D-specta. Previous works have shown [37, 38] that the MAMP influence the composition of secreted furanocoumarins. An influence of the candidate compound on the composition of secreted furanocoumarins upon Pep13 addition might also be possible.

Figure 2a-c shows the culture supernatants of the three reference cultivations (without additives, addition of exclusively 100 μM SA, and addition of exclusively 50 pM Pep13) after 120 h cultivation time. Furthermore, Fig. 2d depicts the supernatant of the culture treated with both SA and Pep13.

Compared to the references (Fig. 2a-c), the supernatant of cells treated with SA and Pep13 (Fig. 2d) showed a potentiated fluorescence intensity at about λ_exc_ = 335 nm and λ_em_ = 400 nm. Furthermore, a distinctive shoulder was observed at about λ_exc_ = 330 nm and λ_em_ = 450 nm indicating that the signal results from a mixture of secreted furanocoumarins [39]. Fig. 2e illustrates the relative fluorescence at a wavelength pair of λ_exc_ = 335 nm and λ_em_ = 400 nm for better comparison. Treatment with 100 μM SA and subsequent addition of 50 nM Pep13 (red column) resulted in a 2.3-fold increase in fluorescence intensity compared to the additive signal of the references. The potentiated effect of SA treatment on furanocoumarin secretion and subsequent Pep13 addition has been shown in previous publications [8, 10, 36].

### Influence of SA and Pep13 concentrations on the OTR

For a better understanding of the respiratory response of parsley cell cultures in terms of defense priming, varying concentrations of SA and Pep13 were applied. The two OTR curves shown in Fig. 1 greatly match. Subsequently, single measurements are shown for the experiments. For better comparability of the measurements between experiments throughout this work, the untreated culture and the culture treated with 100 μM SA and subsequently challenged with 50 pM Pep13 were conducted as reference cultivations in each experiment. Hence, the biological variation was considered for the independent experiments. The OTR at identical experimental conditions of different individual RAMOS experiments, however, show slight differences due to variations in the physiological state of the maintained culture used for inoculation.

**Fig. 3:**
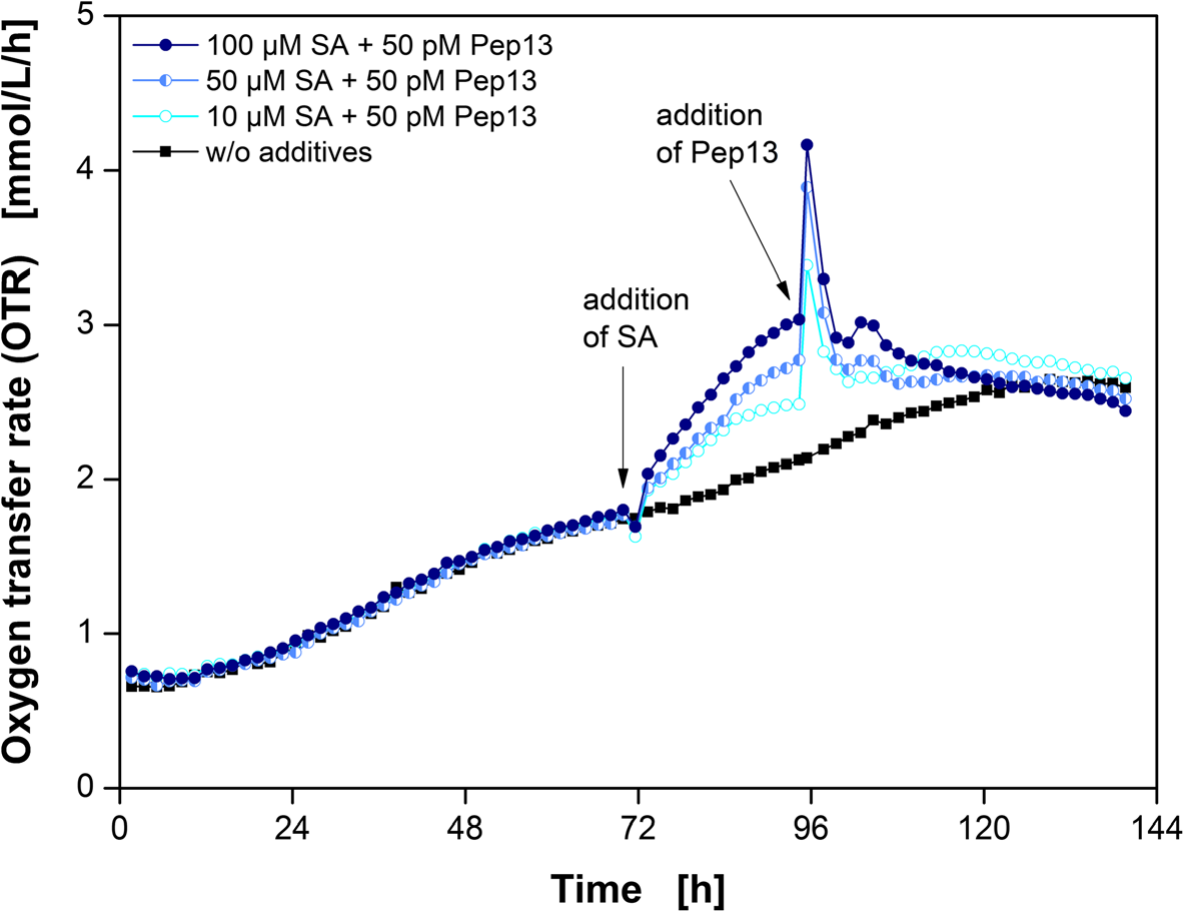
Respiratory responses of parsley cell suspension cultures dependent on the SA concentration. Oxygen transfer rate (OTR) as a function of time for three salicylic acid (SA) concentrations and a reference without additives *(black squares)*. 10 μM SA and 50 pM Pep13 *(open light blue circles)*, 50 μM SA and 50 pM Pep13 *(left half blue circles)*, and 100 μM SA and 50 pM Pep13 *(closed dark blue diamonds)*. Arrows indicate the addition of SA after 72 h and Pep13 after 96 h. Cultivation conditions: 250 mL flask volume, 50 mL filling volume, 180 rpm shaking frequency, 50 mm shaking diameter, and 25 °C

First, the SA concentration was varied and the Pep13 concentration was kept constant at 50 pM. In addition to the standard experiment with 100 μM SA and 50 pM Pep13, Fig. 3 illustrates the OTR of two other priming experiments with 10 μM and 50 μM SA. An untreated parsley culture serves as a reference.

Both a noticeable increase in the OTR after SA addition and two OTR peaks after 50 pM Pep13 treatment were visible, as already shown in Fig. 1. The lower the concentration of SA used, the smaller was the increase in OTR. Consequently, the OTR burst induced by Pep13 addition was also less pronounced after pretreatment with a lower concentration of SA. However, in all three batches the primary peak induced by Pep13 addition exceeded the one that occurred after exclusive Pep13 addition (Additional file 1). This observation agrees with findings of Kauss et al. [25] who detected a correlation between the extracellular H_2_O_2_ concentration after elicitation and the SA concentration used for priming.

A greater change in SA concentration in a different experiment, however, showed the limit of this experimental setup. As little as 1 μM SA did not cause any detectable change in the OTR and Pep13 did not potentiate the OTR burst (Additional file 2A). Since the fluorescence measurements also did not show a potentiation (Additional file 2D), this finding indicates that a minimum level of SA is necessary to activate a detectable primed state in parsley cells. The treatment of cells with 200 μM SA did not lead to a further increase in the OTR compared to the OTR upon addition of 100 μM SA. In contrast, the relative furanocoumarin fluorescence of parsley cells treated with 200 μM SA increased, compared to the cells pretreated with 100 μM SA. This result suggests that the increase in OTR is most likely due to priming since a direct activation of defense responses would probably be stronger upon addition of higher SA concentrations as it is for other defense markers [40]. The OTR burst also occurred upon Pep13 addition when pretreating cells with 100 μM and 200 μM SA (Additional file 2). In tobacco cell cultures, a sigmoidal correlation of SA concentration on catalase activity, and thus an inhibitory effect have been demonstrated, suggesting that significant inhibition of catalase activity requires at least 200 μM SA [27]. However, in parsley cell cultures, 10 μM SA increased the OTR and potentiated furanocoumarin synthesis (Fig. 2, Additional file 1). This might be due to subcellular distribution and -storage of SA, or species specificity. The immediate SA response of the respiratory activity in parsley cells is dose-dependent.

**Fig. 4:**
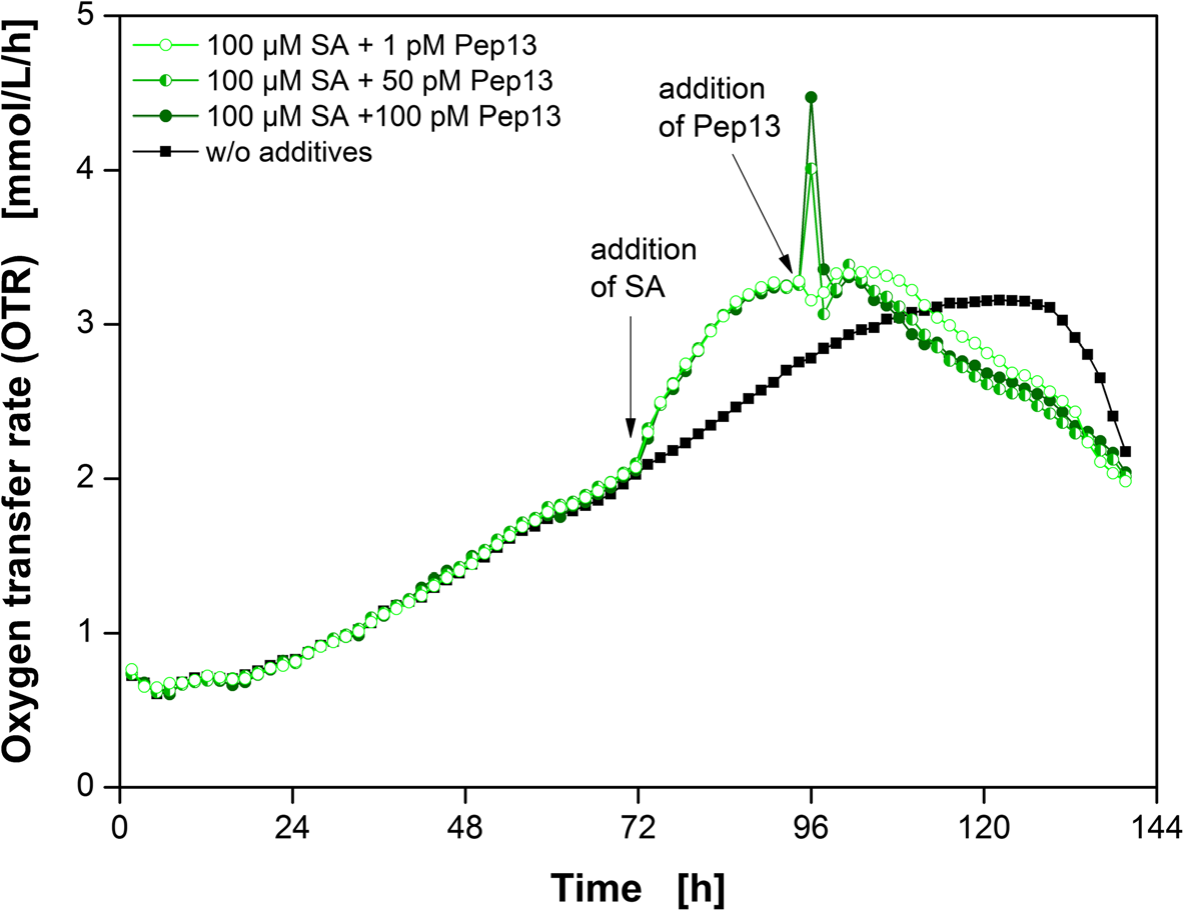
Pep13 dose-dependent respiratory responses of parsley cell suspension cultures. Oxygen transfer rate (OTR) as a function of time for three Pep13 concentrations and a reference without additives *(black squares)*. Addition of 100 μM SA and 100 pM Pep13 (*closed dark green circles*), 100 μM SA and 50 pM Pep13 (*left half green circles*), and 100 μM SA and 1 pM Pep13 *(open light green circles)*. Arrows indicate the addition of SA and Pep13. Cultivation conditions: 250 mL flask volume, 50 mL filling volume, 180 rpm shaking frequency, 50 mm shaking diameter, and 25 °C

**Fig. 5:**
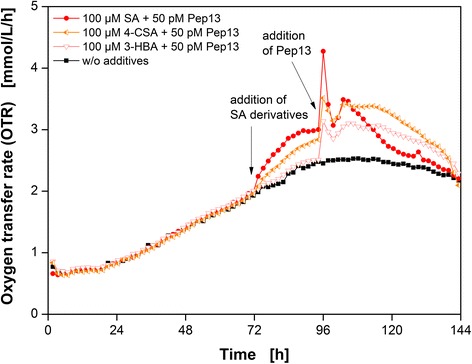
Respiratory response of parsley cell suspension cultures after treatment with SA derivatives and Pep13. Oxygen transfer rate (OTR) as a function of time for three salicylic acid (SA) derivatives and one reference cultivation without additives (*black squares*). 100 μM salicylic acid (*closed red circles*), 100 μM 4-chlorosalicylic acid (*left half orange left triangles*), and 100 μM 3-hydroxybenzoic acid (*open light red down triangles*). Cultivation conditions: 250 mL flask volume, 50 mL filling volume, 180 rpm shaking frequency, 50 mm shaking diameter, and 25 °C. Arrow indicates the addition of the SA derivatives and Pep13

The impact of different Pep13 concentrations on respiratory activity in SA-primed parsley cells was also investigated (Fig. 4). In these experiments, the SA concentration was kept constant at 100 μM. Priming experiments with 100 μM SA and 1 pM, 50 pM and 100 pM Pep13 were performed as illustrated in Fig. 4. An untreated culture served as reference cultivation. Again, SA addition resulted in an increase in the OTR (Figs. 1, 3, 4 and 5). The respiratory response of parsley cells correlated with the Pep13 concentration used. After addition of 100 pM Pep13, the OTR showed a prominent OTR burst and a small secondary peak. A lower concentration of 50 pM SA resulted in a less pronounced OTR burst, but the secondary peak was more pronounced compared to the one occurring after the addition of 100 pM Pep13 (Fig. 4). The addition of only 1 pM Pep13 caused no OTR burst, but the secondary peak was more prominent. However, compared to the OTR of the culture exclusively treated with 100 μM SA, this increase was rather marginal (Additional file 3). As discussed before, the first OTR peak induced by Pep13 might represent an unspecific response, whereas the second one indicates a specific response [33, 34]. The immediate response of the cells was greater with higher Pep13 concentrations. Additionally, the greater the primary OTR burst, the smaller the secondary response. If the OTR burst correlates with an increase in H_2_O_2_ concentration, this phenomenon can be explained by the influence of H_2_O_2_ that can be cytotoxic [33]. The decrease in OTR after a pronounced OTR burst is greater than the decrease in OTR when only a little burst occurred (Fig. 4). The negative influence of the OTR burst on the respiration activity supports the hypothesis that H_2_O_2_ accumulated. If the synthesis of H_2_O_2_ accounts for the OTR burst, the increased effect on cell viability could be the reason for lower concentration of secreted furanocoumarin at higher Pep13 concentrations (Additional file 3).

In summary, the SA and Pep13 concentrations affect respiratory activity and furanocoumarin synthesis. Since the occurrence of an OTR increase after SA addition correlates with the furanocoumarin fluorescence measurements, the OTR increase seems to be a suitable indicator of priming activity.

### Influence of SA derivatives on the OTR

In the previous sections, a correlation between the increase in the OTR of SA-pretreated parsley cells and the potentiation of furanocoumarin secretion was demonstrated. If an increase in the OTR is a measure of priming activity, the expectation is that compounds inducing defense priming would, while priming-negative compounds would not induce an increase in oxygen consumption. Consequently, SA derivatives known as priming-active and priming-inactive compounds were tested for their potential to increase the oxygen consumption in parsley cells.

Figure 5 presents the OTR as a function of time for the priming compounds SA and 4-chloroSA (4-CSA), as well as the priming-negative compound 3-hydroxybenzoic acid (3-HBA). All three cultures were also treated with 50 pM Pep13. An untreated culture served as a reference. In addition, the effect of the priming-inactive compound 4-hydroxybenzoic acid (4-HBA) was investigated (Additional file 4).

Both the addition of 100 μM SA and 100 μM 4-CSA resulted in an increase in the OTR. The increase after the addition of SA was greater than the one after 4-CSA addition. Consequently, parsley cell cultures pretreated with SA and 4-CSA showed potentiated Pep13-activated oxygen consumption. Treatment with the priming-inactive compounds 3-HBA (Fig. 5) or 4-HBA (Additional file 4) did not result in a change in OTR compared to the untreated reference culture. The OTR burst had the same intensity as the OTR burst of the culture exclusively treated with Pep13 (Additional file 4).

The results presented above demonstrate that the addition of priming compounds results in an increase in the OTR, whereas priming-negative compounds do not do so. Conrath et al. [27] revealed that either SA or 4-CSA treated cells showed an inhibitory effect on catalase activity, which was assumed to cause the observed accumulation of ROS. In contrast, priming-inactive SA derivatives such as 3-HBA and 4-HBA did not affect catalase activity [24, 27]. Again, fluorescence intensities were measured to support the OTR results. Pretreatment with SA or 4-CSA potentiated the fluorescence intensity as illustrated in Additional file 4. Treatment with 3-HBA or 4-HBA did not result in potentiation. Thulke et al. [40] demonstrated that the activation of genes in the phenylpropanoid pathway is stronger in parsley cells upon pretreatment with SA or 4-CSA. 3-HBA does not cause this potentiation [40]. The addition of priming-active SA derivatives resulted in a conspicuous increase in the OTR (Fig. 4); the addition of priming-inactive SA derivatives, however, did not have a detectable effect. Our results suggest that an increase in respiratory activity upon addition of a candidate compound can provide reliable information of whether that compound has defense priming-inducing activity in plants.

### Influence of methyl jasmonate and pyraclostrobin on the OTR

**Fig. 6:**
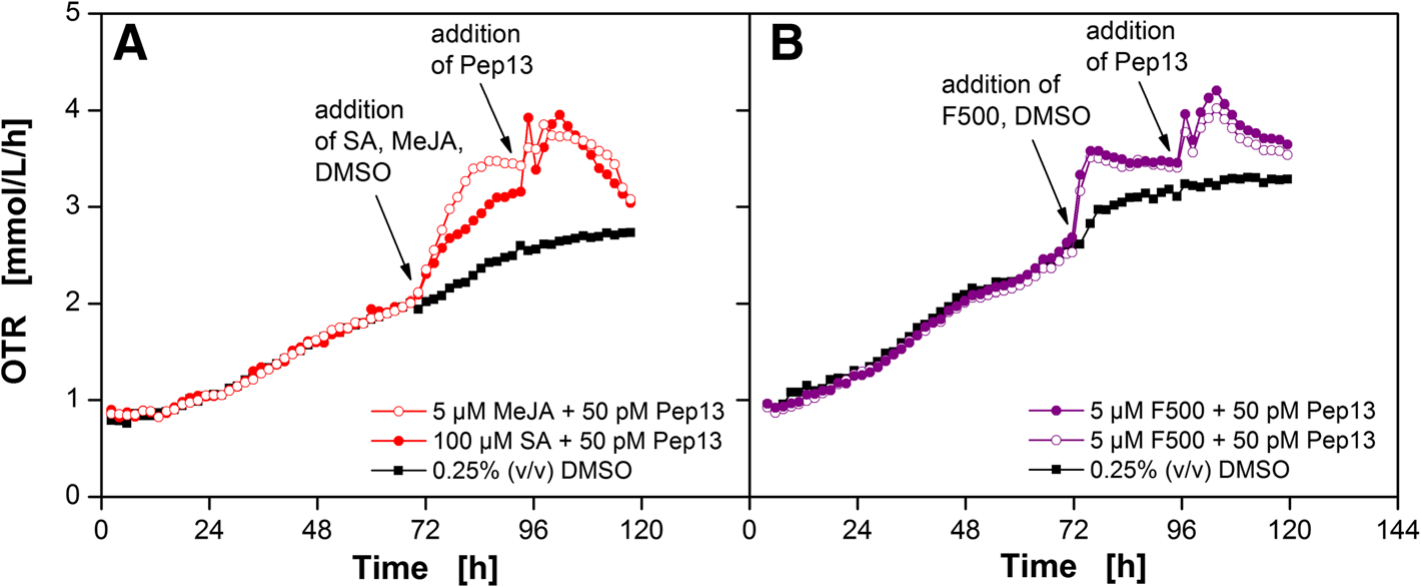
Respiratory response of parsley cell suspension cultures after treatment with MeJA (**a**), F500 (**b**). OTR as a function of time for MeJA + Pep13 (*open red circles*) and F500 + Pep13 (*open and closed red circles*). Both compounds were dissolved in 0.25 % (v/v) DMSO. A parsley cell culture treated with 0.25 % (v/v) DMSO only, and a cell culture treated with 100 μM SA dissolved in DMSO served as a reference. Cultivation conditions: 250 mL flask volume, 50 mL filling volume, 180 rpm shaking frequency, 50 mm shaking diameter, and 25 °C. Arrow indicates the addition of the compounds and Pep13 derivatives

The discrimination between priming-active and priming-inactive compounds based on the OTR was successfully presented for SA and some of its derivatives. Figure 6 depicts the OTR as a function of time for methyl jasmonate (MeJA) (A) and the strobilurin fungicide pyraclostrobin (F500) (B). Both compounds are known to induce priming [41–44]. After addition of MeJA or F500 the OTR increase was greater when compared to the reference cultivation that was treated with DMSO. Thus, the increase in OTR is directly related to the addition of MeJA or F500. The OTR burst after Pep13 addition, however, is smaller as compared to the OTR burst after SA pretreatment and subsequent Pep13 addition. This result shows that compounds addressing a metabolic pathway different than SA [41, 45] still stimulate the respiration activity of parsley cells.

## Conclusions

In this study, we introduced the online measured OTR in shake flasks, as a complimentary technique to the conventional furanocoumarin fluorescence assay in parsley cell cultures. An increase in OTR upon treatment with a candidate compound is consistent with an enhanced Pep13-induced secretion of furanocoumarin phytoalexins. For the parsley cell culture system the RAMOS device provides an immediate, noninvasive, and additional signal for the identification of priming compounds. A fingerprint of the cell culture is obtained by the online measurement of the respiration activity rather than a conventional end point determination of the furanocoumarins. Furthermore, the respiration activity is an overall signal that could be transferred to other plant species. This would also enable the screening for priming compounds for particular crops.

## Methods

### Parsley cell culture

Parsley (*Petroselinum crispum*) cell cultures were grown from callus every 3–4 months in modified Gamborg’s B5 medium [46]. For culture maintenance, 30-40 mL of the suspension culture was cultivated every seven days in 500 mL in baffled shake flasks (one baffle on the lower outside edge, h ≈ 22 mm). Cultures were run with a shaking diameter of 5 cm at 90 rpm and 25 °C in the dark. Experiments were conducted in 250 mL RAMOS shake flasks with a filling volume of 50 mL consisting of 10 mL parsley cell culture and 40 mL fresh modified Gamborg’s B5 medium. The shaking diameter was set to 5 cm and the shaking frequency to 180 rpm. Cultivations were run at 25 °C in the dark.

### Medium and solutions

Parsley cell suspension was cultivated in modified Gamborg’s B5 medium. The micro- and macro-elements, including vitamins, were purchased from DUCHEFA BIOCHEMIE B.V, Haarlem, the Netherlands. The medium was supplemented with 20 g/L sucrose, 20 mg/L 2,4-dichlorophenoxyacetic acid, and 250 mg/L magnesium sulfate heptahydrate. 1 M potassium hydroxide was used to adjust the pH value to 5.5.

Priming-active and priming-inactive SA derivatives were dissolved in distilled water to obtain a stock solution of 10 mM. The pH value was adjusted to 5.5 with a 1 M potassium hydroxide solution. MeJA and F500 were dissolved in DMSO to obtain a stock solution of 5 mM. The compounds were aliquoted and stored at −20 °C. All compound stock solutions were diluted with distilled water or DMSO, respectively, to obtain the required concentration. Compounds were purchased at Sigma-Aldrich Co. LLC. Pep13 was dissolved in water to obtain a 5 nM stock solution which was aliquoted into 1.5 mL microfuge tubes and stored at -20 °C.

### Standard experimental procedure

The procedure consists of two subsequent steps. First, the parsley cell culture was supplemented with 1 mL of a priming-active or priming-inactive SA derivative after 72 h of cultivation time. 125 μL MeJA or F500 solution was added with additionally 875 μL distilled water to add also 1 mL to the parsley cell culture. Then, 1 mL of Pep13 solution was added after 96 h of cultivation time. Finally, a culture sample for furanocoumarin fluorescence determination was taken after 120 h of cultivation time [36].

### Determination of the OTR

The OTR was determined with the respiration activity monitoring system (RAMOS) which was developed and built in-house [12, 13]. This technology enables a quasi-continuous online determination of the oxygen transfer rate (OTR). Conventional shake flasks were equipped with four ports, including a gas inlet and outlet, an inoculation port, and an oxygen sensor for measuring the oxygen partial pressure in the headspace. Throughout the cultivation, rinsing and measuring cycle were repeated continuously. During the rinsing phase, both the inlet and outlet valves were left open to provide aeration of the shake flask. The air flow was adjusted to that in conventional shake flasks with cotton plugs [13]. Prior to the measuring phase, the oxygen sensor was calibrated. During the measuring phase, both valves were closed and, as a result of oxygen consumption, the oxygen partial pressure dropped. The oxygen transfer rate was calculated according to the change in partial pressure over time. Commercial versions of the RAMOS device are available from Kühner AG, Birsfelden, Switzerland and HiTec Zang GmbH, Herzogenrath, Germany.

### Fluorescence measurements

2D-fluorecence measurements were used to determine the secretion of furanocoumarins [47, 48]. Samples were taken from shake flasks cultivated under the same conditions as the RAMOS flasks. The culture broth was harvested after 120 h of cultivation time and centrifuged at 4000 rpm and 4 °C for 10 min. Then, 3 mL of the supernatant was transferred to a quartz cuvette (10x10 mm Suprasil quartz, Hellma GmbH & Co. KG, Müllheim, Germany) and placed in the fluorescence spectrometer (Fluoromax-4, HORIBA Jobin Yvon GmbH, Unterhaching, Germany). A 2D-spectrum from 250 to 500 nm was measured with an increment of 5 nm and a slit width of 1 nm. Data for the bar charts were extracted from the 2D-fluorescence spectra. Fluorescence intensity at an excitation wavelength of 335 nm and an emission wavelength of 400 nm was chosen for comparison [8, 10]. To gain the corrected fluorescence intensity, total fluorescence intensity from the supernatants of the culture was subtracted by the total fluorescence signal of supernatant of the untreated culture. Thereupon, the sum of the corrected fluorescence intensities of the cultures supernatant which were treated exclusively with the priming compound and exclusively with Pep13 was set to 100 % taking the corrected fluorescence intensity in relativity.

## Availability of supporting data

The supporting data of this work is attached as Additional file 1, 2, 3 and 4.

## Additional files

Additional file 1:
**Reference cultivations for the SA-dose dependent responses of parsley cell suspension cultures. **A-C: Oxygen transfer rate (OTR) as a function of time for three salicylic acid (SA) concentrations: (A) addition of 10 μM SA, (B) addition of 50 μM SA, (C) addition of 100 μM SA. Cultivations: w/o additives *(black squares)*, with the addition of exclusively SA after 72 h *(blue diamonds)*, with the addition of exclusively 50 pM Pep13 after 96 h *(gray up triangles)*, and with the addition of SA and 50 pM Pep13 *(blue circles)*. Dotted lines indicate the addition of (1) SA, (2) Pep13, and (3) sampling for the furanocoumarin fluorescence measurements. D-E: The experiments are compared at an excitation wavelength of 335 nm and an emission wavelength of 400 nm. Cultivations: w/o additives *(black columns)*, with the addition of exclusively salicylic acid (SA) *(blue columns)*, with the addition of exclusively 50 pM Pep13 *(gray columns)*, and with the addition of SA and 50 pM Pep13 *(green, light blue and dark blue columns)*. Cultivation conditions: 250 mL flask volume, 50 mL filling volume, 180 rpm shaking frequency, 5 cm shaking diameter, and 25 °C. (TIF 16034 kb) Additional file 2:
**Additional cultivations for the SA-dose dependent responses of parsley cell suspension cultures.** A-C: Oxygen transfer rate (OTR) as a function of time for three salicylic acid (SA) concentrations: (A) addition of 1 μM SA, (B) addition of 100 μM SA, (C) addition of 200 μM SA. Cultivations: w/o additives *(black squares)*, with the addition of exclusively SA after 72 h *(blue diamonds)*, with the addition of exclusively 50 pM Pep13 after 96 h *(gray up triangles)*, and with the addition of SA and 50 pM Pep13 *(purple circles)*. Dotted lines indicate the addition of (1) SA, (2) Pep13, and (3) sampling for the furanocoumarin fluorescence measurements. D-E: The experiments are compared at an excitation wavelength of 335 nm and an emission wavelength of 400 nm. Cultivations: w/o additives *(black columns)*, with the addition of exclusively salicylic acid (SA) *(blue columns)*, with the addition of exclusively 50 pM Pep13 *(gray columns)*, and with the addition of SA and 50 pM Pep13 *(purple columns)*. Cultivation conditions: 250 mL flask volume, 50 mL filling olume, 180 rpm shaking frequency, 5 cm shaking diameter, and 25 °C. (TIF 16270 kb) Additional file 3:
**Reference cultivations for the Pep13-dose dependent responses of parsley cell suspension cultures.** Oxygen transfer rate (OTR) as a function of time for three Pep13 concentrations: (A) addition of 100 pM Pep13, (B) addition of 50 pM Pep13, (C) addition of 1 pM Pep13. Cultivations: w/o additives *(black squares)*, with the addition of exclusively 100 μM SA after 72 h *(blue diamonds)*, with the addition of exclusively Pep13 after 96 h *(gray up triangles)*, and with the addition of 100 μM SA and Pep13 *(green circles)*. Dotted lines indicate the addition of (1) SA, (2) Pep13, and (3) sampling for the furanocoumarin fluorescence measurements. D-E: The experiments are compared at an excitation wavelength of 335 nm and an emission wavelength of 400 nm. Cultivations: w/o additives *(black columns)*, with the addition of exclusively salicylic acid (SA) *(blue columns)*, with the addition of exclusively 50 pM Pep13 *(gray columns)*, and with the addition of 100 μM SA and 50 pM Pep13 *(green columns)*. Cultivation conditions: 250 mL flask volume, 50 mL filling volume, 180 rpm shaking frequency, 5 cm shaking diameter, and 25 °C. (TIF 4013 kb) Additional file 4:
**Reference cultivations for the responses of parsley cell suspension cultures after treatment with SA derivatives and Pep13.** A-D: Oxygen transfer rate (OTR) as a function of time for four salicylic acid (SA) derivatives: (A) salicylic acid (SA), (B) 4-chlorosalicylic acid (4-CSA), (C) 3-hydroxybenzoic acid (3-HBA), and (D) 4-hydroxybenzoic acid (4-HBA). w/o additives *(black squares)*, with the addition of exclusively 100 μM SA derivatives after 72 h *(blue diamonds)*, with the addition of exclusively 50 pM Pep13 after 96 h *(gray up triangles)*, with the addition of 100 μM SA and 50 pM Pep13 *(red circles)*, with the addition of 100 μM 4-CSA and 50 pM Pep13 *(half orange left triangles)*, with the addition of 100 μM 3-HBA and 50 pM Pep13 *(open light red down triangles)*, and with the addition of 100 μM 4-HBA and 50 pM Pep13 *(open brown right triangles)*. Dotted lines indicate the addition of (1) SA, (2) Pep13, and (3) sampling for the furanocoumarin fluorescence measurements. E-H: The experiments are compared at an excitation wavelength of 335 nm and an emission wavelength of 400 nm. Cultivations: w/o additives *(black columns)*, with the addition of exclusively SA derivatives *(blue columns)*, with the addition of exclusively 50 pM Pep13 *(gray columns)*, and with the addition of 100 μM SA derivatives and 50 pM Pep13 *(red columns)*. Cultivation conditions: 250 mL flask volume, 50 mL filling volume, 180 rpm shaking frequency, 50 mm shaking diameter, and 25 °C. (TIF 5309 kb)

## Abbreviations

4-CSA: 4-chlorosalicylic acid
3-HBA: 3-hydroxybenzoic acid
4-HBA: 4-hydroxybenzoic acid
c_SA_: SA concentration
DMSO: Dimethyl sulfoxide
F500: Pyraclostrobin
MAMP: Microbial-associated molecular pattern
MeJA: Methyl jasmonate
OTR: Oxygen transfer rate
PAL: Phenylalanine lyase
RAMOS: Respiration activity monitoring system
ROS: Reactive oxygen species
SA: Salicylic acid
SAR: Systemic acquired resistance
